# Quantitative Iodine-123 single-photon emission computed tomography/computed tomography for Iodine-131 therapy of an autonomously functioning thyroid nodule

**DOI:** 10.1186/s41824-022-00159-w

**Published:** 2023-02-20

**Authors:** Won Woo Lee, Yoo Sung Song, Young So

**Affiliations:** 1 Department of Nuclear Medicine, Seoul National University Bundang Hospital, 82, Gumi-ro 173 Beon-gil, Bundang-gu, Seongnam-si, Gyeonggi-do 13620 Republic of Korea; 2 Department of Nuclear Medicine, Seoul National University College of Medicine, Seoul, Republic of Korea; 3 Institute of Radiation Medicine, Medical Research Center, Seoul National University, Seoul, Republic of Korea; 4 Department of Nuclear Medicine, Konkuk University Medical Center, Seoul, Republic of Korea

**Keywords:** Radioactive iodine, Autonomously functioning thyroid nodule, Toxic nodular goiter, SPECT/CT, Cadmium-zinc-telluride, Quantitation

## Abstract

**Purpose:**

Autonomously functioning thyroid nodules (AFTNs) are treated with iodine-131 (I-131) therapy, which increases the risk of permanent hypothyroidism; however, the risk can be reduced by separately estimating the accumulated activity for the AFTN and extranodular thyroid tissue (ETT).

**Methods:**

A quantitative I-123 single-photon emission computed tomography (SPECT)/CT (5 mCi) was performed in one patient with unilateral AFTN and T3 thyrotoxicosis. The I-123 concentrations measured at 24 h were 12.26 µCi/mL and 0.11 µCi/mL in the AFTN and contralateral ETT, respectively. Thus, the I-131 concentrations and radioactive iodine uptake expected at 24 h by 5 mCi of I-131 were 38.59 µCi/mL and 0.31 for the AFTN and 0.34 µCi/mL and 0.007 for the contralateral ETT. The weight was calculated as CT-measured volume multiplied by 1.03.

**Results:**

In the AFTN patient with thyrotoxicosis, we administered 30 mCi of I-131, which would maximize the 24-h I-131 concentration in the AFTN (226.86 µCi/g) and maintain a tolerable concentration in the ETT (1.97 µCi/g). The percentage of I-131 uptake at 48 h post I-131 administration was 62.6%. The patient achieved a euthyroid state at 14 weeks and maintained the state until 2 years post I-131 administration with an AFTN volume reduction of 61.38%.

**Conclusion:**

The pre-therapeutic planning of quantitative I-123 SPECT/CT may enable a therapeutic window for I-131 therapy, which directs optimal I-131 activity to effectively treat AFTN while preserving the normal thyroid tissue.

**Supplementary Information:**

The online version contains supplementary material available at 10.1186/s41824-022-00159-w.

## Key points

- Question: Is it feasible for I-123 SPECT/CT to be used for planning of I-131 therapy in an autonomously functioning thyroid nodule (AFTN)?
- Pertinent findings: The radioactive iodine uptake of the AFTN and extranodular normal thyroid tissue can be accurately estimated using the I-123 quantitative SPECT/CT.
- Implications for patient care: Patient-tailored I-131 therapy can be possible using the pre-therapeutic I-123 SPECT/CT-based planning in patients with AFTN.

**Supplementary Information:**

The online version contains supplementary material available at 10.1186/s41824-022-00159-w.

## Introduction

Radioactive iodine (RAI) is an effective treatment for hyperthyroidism with a high remission rate and few adverse effects. However, the therapeutic activity of I-131 is still empirically determined in various hyperthyroid diseases (Ross et al. 2016). Patient-tailored dosimetries, including Quimby-Marinelli approach, have also been investigated (Marinelli et al. 1948), but the evaluation of both I-131 uptake (i.e., 24-h RAIU) and retention (i.e., effective half-life) remains challenging; hence, only the 24-h RAIU is widely employed as the key parameter for assessing the response to I-131 therapy in patients with hyperthyroid diseases (Wesche et al. 2001; Krohn et al. 2014). Among hyperthyroid diseases that require I-131 therapy, the autonomously functioning thyroid nodule (AFTN) has peculiar characteristics that differ from those of Graves’ disease. For instance, the required therapeutic activity of I-131 for AFTN is usually higher than that for Graves’ disease due to the lower RAIU (Ross et al. 1984), increasing the risk of normal thyroid destruction and subsequent permanent hypothyroidism. However, in some cases, the permanent hypothyroidism could be avoided due to the significantly lower RAIU of suppressed extranodular thyroid tissue (Nygaard 1998). If the I-131 activity is reduced to avoid permanent hypothyroidism, the inadequate treatment of the AFTN is inevitable. Hypothyroidism is a therapeutic target of RAI treatment in patients with Graves’ disease, while euthyroidism may be achieved by a meticulous approach that balances AFTN treatment and extranodular thyroid damage prevention.

In hyperthyroid diseases, the pretherapeutic planning for I-131 therapy can be performed using I-123 SPECT/CT (Nygaard et al. 2002; Gandhi et al. 2016). Since I-123 emits low-energy (159 keV) photons ideal for gamma camera imaging such as SPECT, the 24-h RAIU can be accurately measured using the quantitative SPECT. Furthermore, CT is an accurate tool for measuring the thyroid volume, which can help determine the AFTN’s size.

The recently developed SPECT/CT scanner made of a cadmium-zinc-telluride (CZT) semi-conductor detector has several advantages compared with the conventional scintillator SPECT/CT scanner. The sensitivity (~ 10 times) and spatial/energy resolution (~ 2 times) were substantially higher in the former compared with that in the latter (Gambhir et al. 2009). Furthermore, the count rate performance remained linear up to ~ 50 mCi of low-energy gamma photons (Bocher et al. 2010), thereby guaranteeing accurate measurement without dead-time count loss in cases of high I-123 accumulation in small lesions such as an AFTN. The quantitative SPECT/CT technology for similar energy (140 keV) photons of Tc-99m has already been applied in a variety of medical conditions (Lee and Group 2019). Thus, quantitative SPECT/CT using CZT detectors is promising for the I-123-based pre-therapeutic planning of I-131 therapy.

We conducted a pre-therapeutic quantitative I-123 CZT SPECT/CT in a patient with an AFTN to estimate the I-131 concentrations in the AFTN and contralateral thyroid tissue. This study aimed to determine the optimal activity of I-131 that would effectively eradicate the AFTN and prevent permanent hypothyroidism.

## Materials and methods

### Patient

**Fig. 1:**
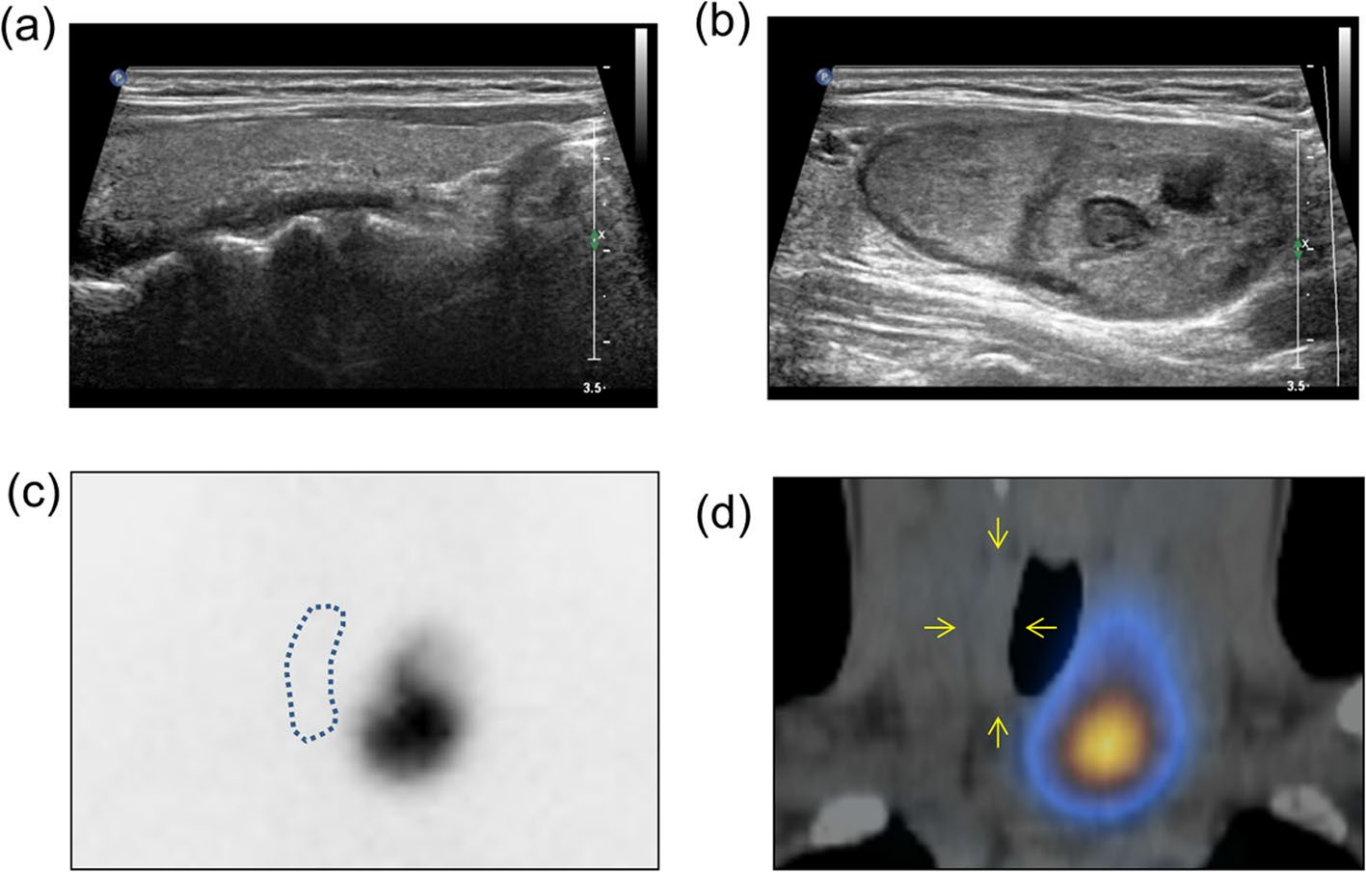
An autonomously functioning thyroid nodule. The neck ultrasound does not show abnormal findings in the right lobe (**a**), while a large thyroid nodule (3.44 × 2.14 × 4.84 cm) is observed in the left thyroid lobe (**b**). Tc-99m pertechnetate scan reveals a hot nodule in the left lobe, while the right lobe is suppressed, as demonstrated on the planar scan (dotted line) (**c**) and SPECT/CT (small arrows) (**d**). *SPECT* single-photon emission computed tomography

A 53-year-old female patient presented to the department of nuclear medicine outpatient clinic of Seoul National University Bundang Hospital in January 2020 due to an exophytic thyroid nodule. She underwent thyroid ultrasonography that revealed a large nodule (3.44 × 2.14 × 4.84 cm) in the left thyroid lobe. A benign follicular nodule was confirmed through fine needle aspiration cytology. No specific abnormality was observed on the right lobe (Fig. 1a, b). On planar scan and SPECT/CT (NMCT670, GE) using Tc-99m pertechnetate (5 mCi), a hot nodule was observed in the left thyroid lobe, while the right thyroid lobe was not visualized (Fig. 1c, d). The percentage of thyroid uptake of the hot nodule found on the left lobe increased to 4.70%, whereas that of the right lobe decreased to 0.04% (normal range 0.78 ± 0.5%) (Lee et al. 2016). Details of the Tc-99m pertechnetate scan and SPECT/CT procedure are provided in the Additional file 1. The thyroid hormone profile assessed via radioimmunoassay was consistent with T3 thyrotoxicosis (Additional file 1: Table S1). The patient provided an informed consent to undergo the therapeutic approach. The study was approved by the institutional ethical review board.

### Pre-therapeutic I-123 quantitative SPECT/CT using a CZT scanner

First, we determined the system sensitivity of the SPECT/CT scanner (NMCT870 CZT, GE), which was reported to be 138.0 cpm/µCi after conducting three independent phantom experiments (Additional file 1: Table S2); this value corresponds to the cross-calibration factor between the CZT scanner and the dose calibrator. The phantom study procedure is also described in the Additional file 1.

The I-123 SPECT/CT was performed in the patient who did not take any medications such as antithyroid drugs or beta-blockers. Following the institutional guideline, she was instructed to restrict iodine intake for 2 weeks before the I-123 SPECT/CT. I-123 (sodium-iodide solution, KIRAMS, 5.1 mCi) was administered to the patient. After ingestion, the remnant I-123 activity (0.1 mCi) was measured. A dose calibrator (CRC-15R, CAPINTEC) was used for the accurate measurement of radionuclide activity. Quantitative SPECT/CT using the CZT scanner (NMCT870 CZT, GE) was performed in the neck area at 1 h, 4 h, and 24 h post I-123 administration. The SPECT/CT protocol is described in the Additional file 1.

The absolute concentration and uptake fraction of I-123 were measured in the thyroid nodule and contralateral thyroid tissue using the vendor-provided quantitative software (Q.Volumetrix MI, GE). The volume of the thyroid nodule and contralateral thyroid tissue was also measured using CT.

## Results

### Measurement of I-123 RAIU in the AFTN and normal thyroid

**Fig. 2:**
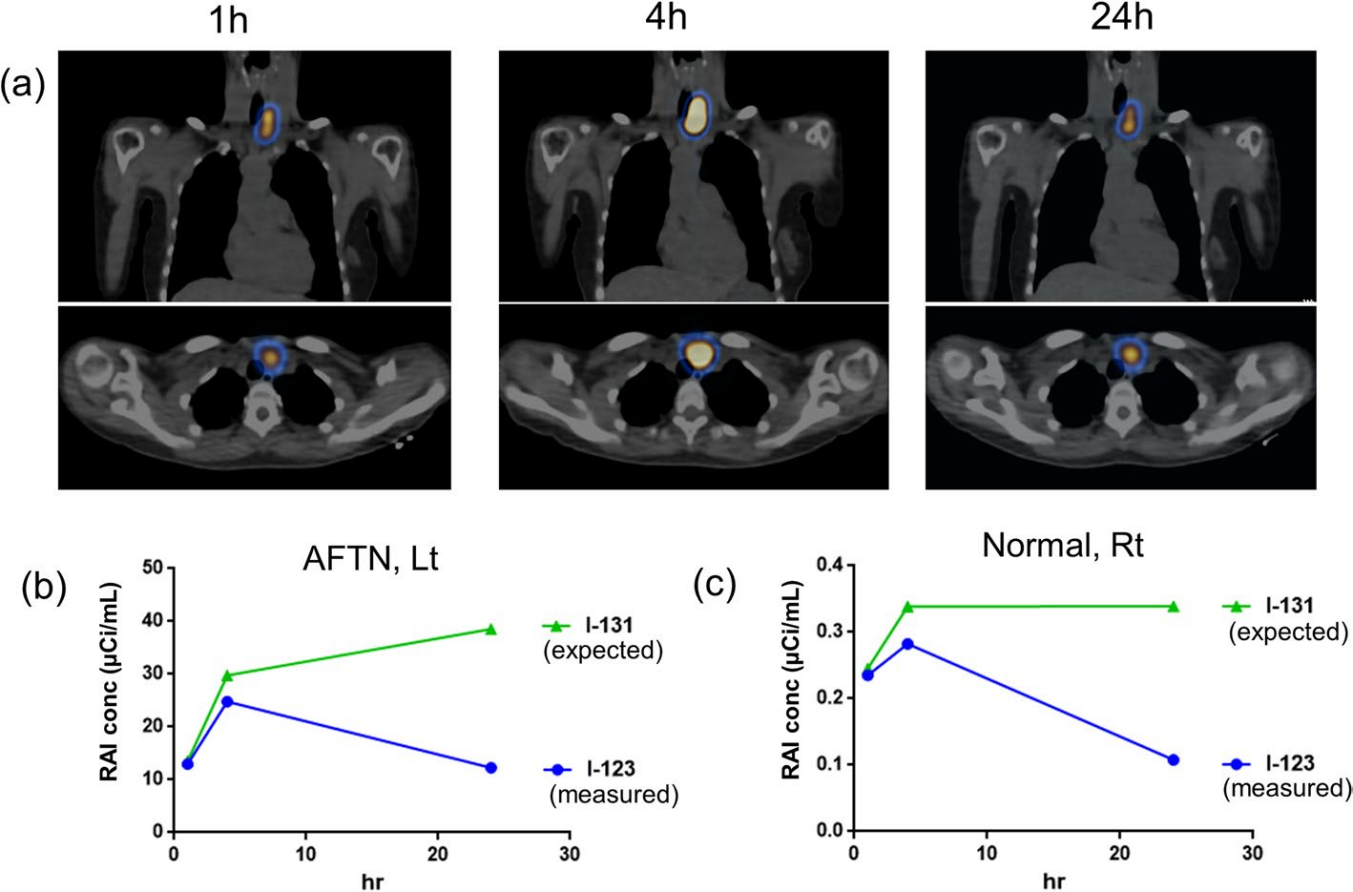
Quantitative I-123 SPECT/CT. Coronal (upper row) and transaxial (bottom row) SPECT/CT images at 1 h, 4 h, and 24 h post I-123 administration (**a**). The measured concentration of I-123 (blue) and expected concentration of I-131 (green) for the hot nodule in the left lobe (**b**) and the contralateral right thyroid lobe (**c**). *RAI* radioactive iodine, *SPECT* single-photon emission computed tomography

The AFTN in the left lobe had the highest uptake at 4-h I-123 SPECT/CT (Fig. 2a). The quantitative SPECT/CT (Q.Volumetrix MI, GE) was used to measure the I-123 concentrations (12.99 µCi/mL, 24.82 µCi/mL, and 12.26 µCi/mL) and the I-123 uptake fractions (0.10, 0.20, and 0.10) at 1-h, 4-h, and 24-h SPECT/CT, respectively (blue in Fig. 2b). The contralateral right lobe showed faint uptake on all the SPECT/CT studies (Fig. 2a); the I-123 concentrations were 0.24 µCi/mL, 0.28 µCi/mL, and 0.11 µCi/mL, while the I-123 uptake fractions were 0.0005, 0.0006, and 0.0002 at 1-h, 4-h, and 24-h SPECT/CT, respectively (blue in Fig. 2c).

### Estimation of the I-131 RAIU in the AFTN and normal thyroid

For the estimation of I-131 concentration, the measured concentration of I-123 (blue in Fig. 2b, c) was initially decay-corrected using the I-123’s half-life of 13.2 h and then reverse decay-corrected using the I-131’s half-life of 192.0 h, both from injection time to measurement time. The equation used in the estimation of I-131 concentration was as follows:

1 $${\text{I-131 radioactivity}} = {\text{I-123 radioacitivity}} \times e^{{\left( {\ln \left( 2 \right) \times \frac{{\text{Measurement time }}}{13.2}} \right)}} \times e^{{\left( { - \ln \left( 2 \right) \times \frac{{\text{Measurement time }}}{192.0}} \right)}}$$

Assuming that 5 mCi of I-131 was administered, the expected I-131 concentrations of the AFTN were calculated as 13.56 µCi/mL, 29.78 µCi/mL, and 38.59 µCi/mL, while the uptake fractions were 0.11, 0.14, and 0.31 at 1 h, 4 h, and 24 h, respectively (green in Fig. 2b), thus suggesting that I-131 was not rapidly metabolized (Silberstein et al. 2012). On the contrary, the expected I-131 concentrations in the normal right thyroid lobe were 0.25 µCi/mL, 0.34 µCi/mL, and 0.34 µCi/mL, while the uptake fractions were 0.0005, 0.0007, and 0.0007 at 1 h, 4 h, and 24 h, respectively (green in Fig. 2c).

### Determination of I-131 activity for AFTN therapy

The I-131 activity for therapy was calculated from the estimated uptake fraction of I-131 at 24 h post administration (i.e., 24-h RAIU of I-131). Weight was calculated as the volume × 1.03.

2 $${\text{I-131 activity}}\; \left( {{\text{mCi}}} \right) = \frac{{{\text{Target concentration of I-131}} \times {\text{weight}}\;\left( {\text{g}} \right)}}{{{\text{24 h uptake fracton of I-131}}}}$$

**Fig. 3:**
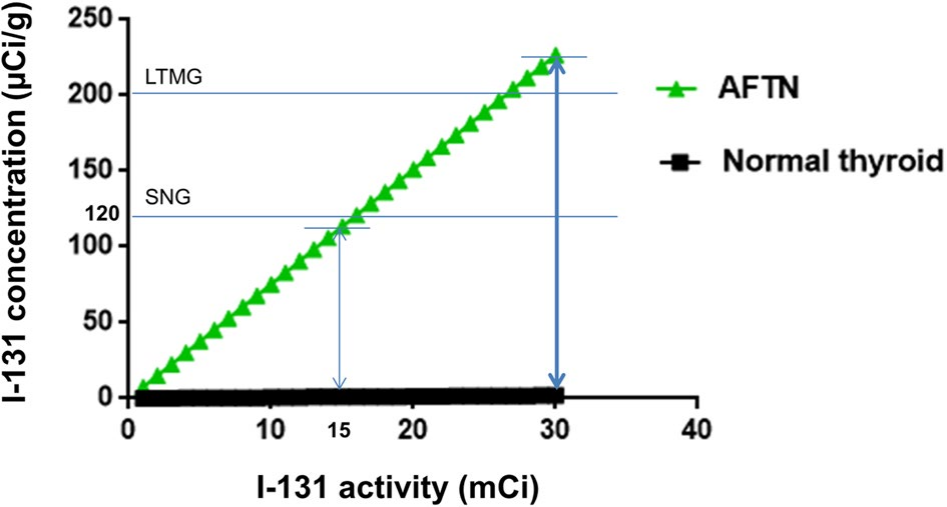
Pre-therapeutic planning for I-131 activity. The target concentration of I-131 was plotted against I-131 activity for the AFTN and the contralateral normal thyroid tissue. The therapeutic windows for the AFTN and normal thyroid tissues are noted when 15 mCi (thin vertical double arrow) and 30 mCi (thick vertical double arrow) are applied. SNG refers to sporadic nontoxic nodular goiter, which was treated with an I-131 concentration of 120 µCi/g (3). LTMG stands for large toxic multi-nodular goiter, which was successfully treated with a target I-131 concentration of 200 µCi/g (14). *AFTN* autonomously functioning thyroid nodule

The expected 24-h RAIUs of I-131 were 0.31 and 0.0007 for the AFTN and normal thyroid, respectively. The weight measurements were 41.0 g for the AFTN and 10.5 g for the normal thyroid. The relationship between I-131 activities and I-131 target concentrations in the AFTN and contralateral normal thyroid tissue was simulated (Fig. 3). For the AFTN, if 15 mCi of I-131 is administered, the expected concentration of I-131 would be 113.4 µCi/g, which is lower than the 120 µCi/g for sporadic nontoxic nodular goiter (SNG) with a 44% size-reduction rate (Wesche et al. 2001). If the I-131 activity is increased to 30 mCi, the AFTN would have 226.86 µCi/g of I-131 concentration, which is greater than the 200 µCi/g suggested for large toxic multi-nodular goiter (LTMG); the reported outcome was a symptomatic improvement rate of 78% after just one course of I-131 therapy (Hamburger and Hamburger 1985). On the contrary, the expected I-131 concentrations in the normal thyroid tissue would be 0.99 µCi/g if 15 mCi of I-131 is administered and 1.97 µCi/g if 30 mCi of I-131 is administered. Therefore, we decided to administer 30 mCi of I-131 to the patient whose risk of permanent hypothyroidism was expectedly low (Krohn et al. 2014).

### I-131 therapy and post-therapeutic I-131 uptake using a rate meter

Four weeks later, the patient was subjected to a low iodine diet for 2 weeks, and 30 mCi of I-131 was administered. After 48 h, the percentage of I-131 uptake was 62.7% using a thyroid uptake system (Koroid, SeYoung NDC, Ltd, Seoul, Korea). The measurement procedure is described in the Additional file 1.

### Treatment response

At 14 weeks post-therapy, the patient felt that the thyroid nodule was no longer exophytic. The follow-up neck ultrasonography at 30 weeks and 2 years post I-131 therapy demonstrated volume reduction of 48.52% and 61.38%, respectively, from 18.67 mL (3.44 × 2.14 × 4.84 cm) to 9.61 mL (2.92 × 1.54 × 4.08 cm) to 7.21 mL (2.47 × 1.43 × 3.90 cm). The volume was calculated through an ultrasonographic examination using an equation for ovoids: volume = width × depth × length × 0.524 (Malago et al. 2008). The T3 thyrotoxic state prior to therapy returned to a euthyroid state at 14 weeks and was maintained until 2 years post I-131 therapy (Additional file 1: Table S1). No medications were required during the follow-up period.

## Discussion

The principle of the current study lies on the separate estimation of the 24-h RAIU of I-131 for the AFTN and normal tissue to effectively treat the AFTN without leading to permanent hypothyroidism. Thus, the concept for a successful treatment of an AFTN needs to be different from that of Graves’ disease. In Graves’ disease, permanent hypothyroidism is a therapeutic goal rather than an adverse effect (Ross et al. 2016); in an AFTN, cure without the development of permanent hypothyroidism can be achieved if the suppressed extranodular thyroid tissue is not damaged by I-131 therapy (Ross et al. 1984). Therefore, a high-resolution imaging modality is required for the pre-therapeutic planning of an AFTN. However, previous studies were mainly conducted using less accurate modalities such as a rate meter, recti-linear scanner, or planar gamma imaging (Wesche et al. 2001; Nygaard et al. 1993). The AFTN was not differentiated from extranodular thyroid tissue in the aforementioned studies wherein only the 24-h RAIU was obtained without determining where the I-131 accumulated. As a result, the reported incidence of permanent hypothyroidism after I-131 administration for the AFTN varied between 7.6 and 60% (Berglund et al. 1991; Ceccarelli et al. 2005). Published guidelines simply recommend prescribing sufficient activity of I-131, which is usually greater than that for Graves’ disease owing to the lower 24-h RAIU of the AFTN (Ross et al. 2016). As a result, non-toxic AFTN patients without any medications at the beginning become permanently hypothyroid after I-131 treatment, which requires life-long thyroid hormone replacement.

With a target I-131 concentration of 226.86 µCi/g for the AFTN, a total radiation dose of more than 100 Gy should be delivered to this area, which was usually accomplished by administering 100–150 µCi/g of I-131 (Hermus and Huysmans 1998; Hegedus et al. 2003). In fact, the target concentration to the AFTN (226.86 µCi/g) in the current study was the highest compared with those in previous studies, which recommended 200 µCi/g at most (Ross et al. 2016; Wesche et al. 2001; Nygaard et al. 1993).

The target was not only the AFTN but also the extranodular thyroid tissue in the current study. The expected I-131 concentration in the contralateral normal thyroid tissue was only 1.97 µCi/g, which will not cause serious harm to the suppressed thyroid tissue (Krohn et al. 2014). The patient’s clinical response suggested that the extranodular thyroid tissue was saved until 2 years post I-131 administration.

### Limitations

Since this study is a single case report with an unprecedented approach, the results cannot be generalized to other populations. Furthermore, post-therapeutic dosimetry was not performed in this study. The clinical response was only examined to ascertain the integrity of the pre-therapeutic planning. However, it is impossible to quantitatively and separately assess the I-131 uptake post therapy in the AFTN and extranodular normal thyroid. We just measured 48-h RAIU as 62.6% as a whole. In this regard, quantitative I-131 SPECT/CT warrants further investigation in the future.

## Conclusion

Quantitative I-123 SPECT/CT using a CZT scanner shows a promising potential for the pre-therapeutic planning of the I-131 therapy for an AFTN. The effective treatment of AFTN with extranodular thyroid tissue preservation may be achieved through a meticulous therapeutic window approach using the I-123 quantitative CZT SPECT/CT.

## Supplementary Information

**Additional file 1.** Tc-99m Pertechnetate Scan and Quantitative Single-photon Emission Computed Tomography/Computed Tomography (SPECT/CT). I-123 Phantom Study for System Sensitivity of Cadmium-zinc-telluride (CZT) Scanner. I-123 Quantitative SPECT/CT. Measurement of I-131 Uptake Post Therapy. Details of acquisition/reconstruction protocols.

## Data Availability

The datasets used and/or analyzed during the current study are available from the corresponding author on reasonable request.

## References

[CR1] Berglund J, Christensen SB, Dymling JF, Hallengren B (1991). The incidence of recurrence and hypothyroidism following treatment with antithyroid drugs, surgery or radioiodine in all patients with thyrotoxicosis in Malmo during the period 1970–1974. J Intern Med.

[CR2] Bocher M, Blevis IM, Tsukerman L, Shrem Y, Kovalski G, Volokh L (2010). A fast cardiac gamma camera with dynamic SPECT capabilities: design, system validation and future potential. Eur J Nucl Med Mol Imaging.

[CR3] Ceccarelli C, Bencivelli W, Vitti P, Grasso L, Pinchera A (2005). Outcome of radioiodine-131 therapy in hyperfunctioning thyroid nodules: a 20 years' retrospective study. Clin Endocrinol (oxf).

[CR4] Gambhir SS, Berman DS, Ziffer J, Nagler M, Sandler M, Patton J (2009). A novel high-sensitivity rapid-acquisition single-photon cardiac imaging camera. J Nucl Med.

[CR5] Gandhi A, Wong KK, Gross MD, Avram AM (2016). Lingual thyroid ectopia: diagnostic SPECT/CT imaging and radioactive iodine treatment. Thyroid.

[CR6] Hamburger JI, Hamburger SW (1985). Diagnosis and management of large toxic multinodular goiters. J Nucl Med.

[CR7] Hegedus L, Bonnema SJ, Bennedbaek FN (2003). Management of simple nodular goiter: current status and future perspectives. Endocr Rev.

[CR8] Hermus AR, Huysmans DA (1998). Treatment of benign nodular thyroid disease. N Engl J Med.

[CR9] Krohn T, Hanscheid H, Muller B, Behrendt FF, Heinzel A, Mottaghy FM (2014). Maximum dose rate is a determinant of hypothyroidism after 131I therapy of Graves' disease but the total thyroid absorbed dose is not. J Clin Endocrinol Metab.

[CR10] Lee WW, Group KS (2019). Clinical applications of technetium-99m quantitative single-photon emission computed tomography/computed tomography. Nucl Med Mol Imaging.

[CR11] Lee H, Kim JH, Kang YK, Moon JH, So Y, Lee WW (2016). Quantitative single-photon emission computed tomography/computed tomography for technetium pertechnetate thyroid uptake measurement. Medicine (baltimore).

[CR12] Malago R, D'Onofrio M, Ferdeghini M, Mantovani W, Colato C, Brazzarola P (2008). Thyroid volumetric quantification: comparative evaluation between conventional and volumetric ultrasonography. J Ultrasound Med.

[CR13] Marinelli LD, Quimby EH, Hine GJ (1948). Dosage determination with radioactive isotopes; practical considerations in therapy and protection. Am J Roentgenol Radium Ther.

[CR14] Nygaard B (1998). Changes in the thyroid technetium-99m scintigram after antithyroid and subsequent radioiodine treatment for solitary autonomous nodules. Thyroid.

[CR15] Nygaard B, Hegedus L, Gervil M, Hjalgrim H, Soe-Jensen P, Hansen JM (1993). Radioiodine treatment of multinodular non-toxic goitre. BMJ.

[CR16] Nygaard B, Nygaard T, Court-Payen M, Jensen LI, Soe-Jensen P, Gerhard Nielsen K (2002). Thyroid volume measured by ultrasonography and CT. Acta Radiol.

[CR17] Ross DS, Ridgway EC, Daniels GH (1984). Successful treatment of solitary toxic thyroid nodules with relatively low-dose iodine-131, with low prevalence of hypothyroidism. Ann Intern Med.

[CR18] Ross DS, Burch HB, Cooper DS, Greenlee MC, Laurberg P, Maia AL (2016). 2016 American Thyroid Association guidelines for diagnosis and management of hyperthyroidism and other causes of thyrotoxicosis. Thyroid.

[CR19] Silberstein EB, Alavi A, Balon HR, Clarke SE, Divgi C, Gelfand MJ (2012). The SNMMI practice guideline for therapy of thyroid disease with 131I 3.0. J Nucl Med.

[CR20] Wesche MF, Tiel VBMM, Lips P, Smits NJ, Wiersinga WM (2001). A randomized trial comparing levothyroxine with radioactive iodine in the treatment of sporadic nontoxic goiter. J Clin Endocrinol Metab.

