# Supplementary material for: Quantitative Iodine-123 single-photon emission computed tomography/computed tomography for Iodine-131 therapy of an autonomously functioning thyroid nodule
Source: Eur J Hybrid Imaging. 2023 Feb 20;7:4. doi: 10.1186/s41824-022-00159-w (PMC9939564; doi:10.1186/s41824-022-00159-w)
Supplement: Supplementary file 1 — Additional file 1. Tc-99m Pertechnetate Scan and Quantitative Single-photon Emission Computed Tomography/Computed Tomography (SPECT/CT). I-123 Phantom Study for System Sensitivity of Cadmium-zinc-telluride (CZT) Scanner. I-123 Quantitative SPECT/CT. Measurement of I-131 Uptake Post Therapy. Details of acquisition/reconstruction protocols. [file 41824_2022_159_MOESM1_ESM.docx]

**Tc-99m pertechnetate scan and quantitative single-photon emission computed tomography/computed tomography**

Tc-99m pertechnetate (5 mCi) eluted out of a Mo-99/Tc-99m generator (Samyoung, Korea) was intravenously injected into the patient. Twenty minutes later, conventional SPECT/CT scanner (NMCT670, GE) equipped with low-energy high-resolution collimators was used over 1 min with neck extension to acquire an anterior planar image. Then, SPECT/CT was performed using the continuous acquisition mode for 1 min. The Tc-99m pertechnetate activities before and after the injection were measured using a dose calibrator (CRC-15R, CAPINTEC) cross-calibrated with the SPECT/CT scanner. The system sensitivity was 151.8 cpm/µCi. The SPECT acquisition conditions were as follows: energy window peak at 140 keV (20% window of 126-154 keV) and scatter window peak at 120 keV (10% window of 115-125 keV). The SPECT image was reconstructed using the iterative ordered subset expectation maximisation (OSEM) method with 2 iterations and 10 subsets. The triple corrections for the quantitative SPECT (CT attenuation correction, scatter correction, and resolution recovery) were employed during image reconstruction. The CT acquisition parameters were as follows: tube voltage, 120 kVp; tube current, 30 mA; detector collimation, 20 mm (=16×1.25); helical thickness, 2.5 mm; table speed, 37 mm/s; table feed per rotation, 18.75 mm/rot; tube rotation time, 0.5 s; and pitch, 0.938:1. The CT was reconstructed through adaptive statistical iterative reconstruction (ASiR).

**I-123 phantom study for system sensitivity of cadmium-zinc-telluride scanner**

The following phantom study was independently performed three times (Supplemental Table 2). A uniform cylinder phantom (diameter, 20 cm; length, 30 cm; and weight in empty state, 2.45 kg) was used. First, the phantom was filled with 9.3 L of tap water until the phantom weighed 11.75 kg. Then, I-123 (1 mCi) was injected into the phantom. I-123 was measured using a dose calibrator (CRC-15R, CAPINTEC) calibrated every month based on the National Institute of Standards and Technology traceable standard of Co-57 point source. The phantom was placed at the centre of the field of view and 25 cm away from the individual detectors. Then, SPECT was performed for 1 min using the following parameters: 360-degree coverage using the continuous mode, the peak energy of 159 keV with a 20% (±10%) window (143.1-174.9 keV), the scatter energy of 130 keV with a 20% (±10%) window (117-143 keV), and a zoom factor of 1.0. Then, spiral CT was performed using the following parameters: tube potential, 120 kVp; tube current, 80-220 mA with Smart protocol; beam collimation, 20 mm (=1.25×16); pitch, 1.375:1; speed, 27.5 mm/rotation; and tube rotation time, 0.5 s. SPECT images were iteratively reconstructed using the OSEM algorithm with 4 iterations and 10 subsets. During SPECT reconstruction, CT-based attenuation correction, dual-energy window scatter correction with a weighting factor of 0.61, and correction of depth-dependent collimator-detector response were applied using Q.Volumetrix MI (GE) on a dedicated workstation (Xeleris version 4DR, GE).

**I-123 quantitative SPECT/CT**

The patient was instructed to maintain a low iodine diet for 2 weeks before I-123 SPECT/CT according to the institutional diet guideline. At the time of I-123 intake (5 mCi, sodium iodide solution, KIRAMS), the patient was in a fasting state. The I-123 activity was measured before and after intake using the dose calibrator (CRC-15R, CAPINTEC), which had been cross-calibrated with the CZT SPECT/CT scanner (NMCT870 CZT, GE). The time of measurement was also recorded.

An image of the neck area was obtained using the CZT SPECT/CT scanner equipped with wide-energy high resolution collimators at 1 h, 4 h, and 24 h after I-123 administration. The following acquisition parameters were used: feet-first counter-clockwise rotation, peak energy at 159 keV with a 10% window (142-174 keV), scatter energy window at 130 keV with a 10% window (117-143 keV), step and shoot mode, 10 s acquisition per step, 3° angle step, body contour option, and a zoom factor of 1.28. The reconstruction was performed on Q.Volumetrix MI (Xeleris 4DR, GE) using the following parameters: OSEM with 4 iterations and 10 subsets, triple corrections (CT attenuation correction, dual-energy window scatter correction, and resolution recovery), matrix of 128х128, and slice thickness of 3.45 mm. For the CT, the acquisition parameters were tube voltage of 120 kVp, tube current of 30 mA, detector collimation (16х1.25 = 20 mm), helical thickness of 2.5 mm, table speed of 37 mm/s, table feed per rotation of 18.75 mm/rot, tube rotation time of 0.5 s, pitch of 0.938:1, matrix of 512х512, and slice thickness of 2.5 mm. The CT image was reconstructed using a vendor-provided CT dose reduction technology of ASiR (GE).

**Measurement of I-131 uptake post therapy**

Upon the administration of I-131 (30 mCi), additional 1 mCi of I-131 was kept aside as a reference activity. Forty-eight hours later, the counts of I-131 were measured using a thyroid uptake system (Koroid, SeYoung NDC, Ltd, Seoul, Korea). The reference activity was counted in a thyroid phantom with B-filter for 60 s. Then, the counts were multiplied by 30 (reference counts). The patient’s thyroid counts were measured at the anterior neck with B-filter for 10 s. Then the counts were multiplied by 6 (thyroid counts). The percentage of I-131 uptake was calculated using the following equation:

$$Percentage I­131 uptake=\frac{Thyroid counts}{Reference counts}\times100$$

**Additional file 1: Table S1. Thyroid function test results**

| Tests (normal reference range) | Before therapy | Fourteen weeks post therapy | Thirty weeks post therapy | Two years post therapy |
| --- | --- | --- | --- | --- |
| T3 (81–197 ng/dL) | 222 | 130 | 127 | 139 |
| Free T4 (0.89–1.78 ng/dL) | 1.57 | 0.98 | 1.30 | 1.20 |
| TSH (0.3–4.0 µIU/mL) | <0.05 | 2.01 | 2.67 | 2.71 |
| Anti-TG antibody (0–60 U/mL) | 570 | 161 | 183 | 53 |

T3, triiodothyronine; T4, thyroxine; TSH, thyroid-stimulating hormone; anti-TG, anti-thyroglobulin

**Additional file 1: Table S2. Phantom study results for the system sensitivity of the CZT SPECT/CT* scanner**

| Session | Time (hr) | Activity by dose calibrator (µCi) | Counts by two-headed SPECT/CT (counts/min) | | | System sensitivity (counts/min/µCi) per detector |
| --- | --- | --- | --- | --- | --- | --- |
|  |  |  | AC** | AC and SC† | AC, SC, and RR‡ |  |
| 1 | 0 | 973 | 339,995 | 229,108 | 275,922 | 141.8 |
|  | 8 | 647 | 221,640 | 168,305 | 180,647 | 139.6 |
|  | 24 | 286 | 94,220 | 82,050 | 72,323 | 126.4 |
| 2 | 0 | 1,071 | 364,000 | 238,406 | 294,628 | 137.5 |
|  | 8 | 706 | 242,542 | 181,234 | 198,830 | 140.8 |
|  | 24 | 308 | 101,496 | 88,134 | 80,978 | 131.5 |
| 3 | 0 | 1,070 | 370,777 | 241,104 | 299,682 | 140.0 |
|  | 8 | 690 | 241,597 | 178,183 | 197,384 | 143.0 |
|  | 24 | 311 | 100,762 | 95,225 | 87,470 | 140.6 |
|  | | | | | | 138.0 |

*CZT SPECT/CT, cadmium-zinc-telluride single-photon emission computed tomography/computed tomography; **AC, attenuation correction; ^†^SC, scatter correction; ^‡^RR, resolution recovery
